# A Multi-Classifiers Based Algorithm for Energy Efficient Tasks Offloading in Fog Computing

**DOI:** 10.3390/s23167209

**Published:** 2023-08-16

**Authors:** Moteb K. Alasmari, Sami S. Alwakeel, Yousef A. Alohali

**Affiliations:** College of Computer and Information Sciences, King Saud University, Riyadh 11362, Saudi Arabia; ssalwakeel@ksu.edu.sa (S.S.A.); yousef@ksu.edu.sa (Y.A.A.)

**Keywords:** fog computing, internet of things, module placement, energy efficiency, task offloading, iFogSim

## Abstract

The IoT has connected a vast number of devices on a massive internet scale. With the rapid increase in devices and data, offloading tasks from IoT devices to remote Cloud data centers becomes unproductive and costly. Optimizing energy consumption in IoT devices while meeting deadlines and data constraints is challenging. Fog Computing aids efficient IoT task processing with proximity to nodes and lower service delay. Cloud task offloading occurs frequently due to Fog Computing’s limited resources compared to remote Cloud, necessitating improved techniques for accurate categorization and distribution of IoT device task offloading in a hybrid IoT, Fog, and Cloud paradigm. This article explores relevant offloading strategies in Fog Computing and proposes MCEETO, an intelligent energy-aware allocation strategy, utilizing a multi-classifier-based algorithm for efficient task offloading by selecting optimal Fog Devices (FDs) for module placement. MCEETO decision parameters include task attributes, Fog node characteristics, network latency, and bandwidth. The method is evaluated using the iFogSim simulator and compared with edge-ward and Cloud-only strategies. The proposed solution is more energy-efficient, saving around 11.36% compared to Cloud-only and approximately 9.30% compared to the edge-ward strategy. Additionally, the MCEETO algorithm achieved a 67% and 96% reduction in network usage compared to both strategies.

## 1. Introduction

The use of the Internet of Things (IoT) is becoming increasingly dominant in many areas of daily life, including medical care, industrial automation, smart homes, and emergency response. The interconnectivity of a massive number of devices through the IoT has led to the generation of huge amounts of diverse data, often referred to as data explosions. According to the International Data Corporation (IDC), by 2025, IoT devices connected to the network are expected to exceed 41 billion, producing more than 79 ZB of data yearly [1].

While Cloud computing has become a smart option for IoT device tasks due to its vast data storage and cost-effectiveness, the proposed solution does not provide a broad resolution for challenges, such as real-time demands, latency-sensitive applications, and limited network bandwidth [2]. These issues often arise due to the considerable physical distance between end-users and data centers of Cloud service providers like Amazon Web Services (AWS), Google, ALTUS, Apple, Facebook, TATA, China Unicom Matrix, Microsoft, and Bell [3,4]. Moreover, the exponential growth of data generated by the expanding number of connected IoT devices presents numerous challenges and complications for Cloud computing, including [5]:

1. The first challenge faced by many IoT applications is the requirement of short latency, especially in Internet of Vehicles and industrial applications. These applications, such as drone control and vehicle-to-vehicle communication, require latency of only a few tens of milliseconds;
2. The second challenge is limited link bandwidth; as the number of wirelessly connected IoT devices continues to rise, the wireless spectrum has become overloaded. This overcrowding leads to insufficient bandwidth for transmitting all the data to the Cloud. To address this issue, researchers propose processing the majority of the generated data at the endpoint;
3. Finally, IoT devices face limitations in their data communication capability due to cost and energy limitations. Therefore, device task offloading should consider and effectively consume the available energy in network devices.

Cloud computing has a significant downside due to the distance between the Cloud and the data source, which can cause delays and service performance degradation, especially for time-sensitive applications like video streaming and online gaming [6]. Devices at the thing level are inadequate for most applications as they lack the computational and storage capabilities required by many applications and algorithms. This results in limited functionality, making it infeasible to deploy fully featured applications. Alternatively, applications and algorithms can be hosted on Cloud nodes to perform computationally intensive operations. However, this approach presents issues, including high latency between IoT devices and Cloud nodes, difficulty maintaining connections to remote Cloud sites, and security concerns due to private data being transmitted and processed in remote Cloud sites. On the other hand, Fog devices serve as middle links in the chain, where operational data is processed and stored by small data centers with low latency. These devices connect IoT devices with Cloud nodes, making them a promising option for hosting applications and algorithms. Fog devices offer Cloud-like services in terms of computational power and storage capacity, with low latency and high bandwidth [7,8,9]. To address these issues, researchers are working on improving Cloud computing models to achieve better performance. Additionally, data processing costs in the Cloud are important factors to consider. High charges may discourage users from paying for the service, even if it offers reduced energy consumption and required time [10]. As a solution to these limitations, Fog computing is gaining popularity in handling the computational demands of IoT mobile applications by enabling devices to perform complex tasks at the network edge [11].

In order to control whether tasks should be offloaded to the Fog, sophisticated resource management and scheduling mechanisms are required to efficiently handle Fog devices [11]. However, due to the limited resources of Fog computing compared to remote Cloud, Cloud task offloading is still widespread. Therefore, optimized techniques are required to accurately determine and distribute IoT device task offloading in a hybrid IoT, Fog, and Cloud paradigm. To address this, an intelligent and energy-efficient task offloading algorithm for the IoT, Fog, and Cloud computing paradigm is proposed. This algorithm utilizes a multi-classifier system to calculate the attributes of the task, network, and processing Fog nodes to define the best-enhanced solution for service node selection. The multi-classifier system is designed to improve the offloading request situations, considering energy consumption, data delivery throughput, service request transfer, and execution time.

The main objective of this research is to propose a novel energy-efficient task offloading method for IoT, Fog, and Cloud computing paradigms, using a multi-classifier-based system. The multi-classifier system will provide the best prominent solution that considers all the available attributes of task, network, and processing nodes (alternative IoT, Fog, and Cloud) as input. It will then process these attributes to classify and elect the best node for processing the task considering transfer and execution time, energy consumption, and other service cost parameters. The major contributions of this paper are concise as follows:

- Propose and implement a novel model using multi-classifier machine learning for the problem of task offloading decisions in an IoT Fog–Cloud computing paradigm;
- Conduct an evaluation and validation of the proposed model by comparing it to existing works, demonstrating how the proposed approach enhances Quality of Service (QoS) parameters, precisely application response time, network usage, and energy consumption.

The paper is organized as follows: Section 2 provides an overview of related work. Section 3 presents the system architecture and computation models. The MCEETO algorithm is termed in detail in Section 4, along with simulation evaluation results. The paper concludes with a summary of the findings in the final section.

## 2. Related Works

Recent literature has shown a growing interest in exploring the benefits of Fog computing for IoT environments and applications. Among the challenges faced by IoT services, energy consumption is a key factor that impacts availability, reliability, and quality of service (QoS). This section provides a summary of fresh studies that have proposed energy-efficient approaches for task offloading in the Fog computing paradigm. Gupta et al. [12] proposed a centralized edge-ward module placement algorithm for distributed applications represented as Directed Acyclic Graphs (DAGs). Their algorithm starts module placement from lower-level Fog nodes and moves up the hierarchy until a node with adequate resources is found. However, their approach only supports vertical scaling of modules and does not consider horizontal connections among Fog nodes at the same level.

Kim et al. [13] have proposed joint user equipment and Fog server energy optimization (JUFO) scheme as a substitute for the energy-minimizing partial offloading (EMPO) scheme for optimizing energy consumption in Cloud tasks. The JUFO scheme controls the popularity distribution of Cloud tasks and an energy consumption model to reduce the combined energy usage of the User Equipment (UE) and the Fog server. In contrast, the approach proposed in [14] optimizes the decision-making process for task offloading, Fog node selection, and computation resource allocation. The authors have formulated the task offloading problem as a Mixed-Integer Nonlinear Program (MINLP) and assigned weights to the coefficients for energy and time consumption based on the remaining battery energy of the device and user demands. They have introduced a sub-optimal solution using a hybrid version of GA and PSO. According to the authors, their proposed approach surpasses the performance of the standard baseline Scheme.

In Wang et al.’s [15] study, the authors present a task offloading scheme for multi-pair Fog–RAN systems using massive multiple-input multiple-output (MIMO) to achieve energy efficiency. The scheme objects to minimize energy consumption by considering realistic imperfect channel state information (CSI) and addressing a non-convex problem involving joint power allocation and task offloading. To tackle this problem, the authors propose a two-step iterative sequential optimization framework. In the first step, computation resources and tasks are allocated based on a given power allocation, while the second step determines the power allocation to minimize energy usage. Simulation results validate that the proposed approach significantly reduces energy consumption compared to benchmark schemes.

In another study by Cai et al. [16], they introduce the JOTE (Joint Offloading of Tasks and Energy) algorithm for Fog-enabled IoT networks. This algorithm focuses on minimizing task delay and energy consumption for a specific task, assuming the absence of task queues. The authors debate that joint offloading of energy and task bits becomes more beneficial as the number of helper nodes increases. Furthermore, to address energy consumption and task execution delay in the presence of task queues, an online offloading policy based on Lyapunov optimization is developed. Numerical experimental results prove that JOTE effectively reduces task delay in Fog-enabled IoT networks.

Keshavarznejad et al. [17] have proposed metaheuristic algorithms, namely NSGA II and Bees, to improve both the probability of offloading and the energy consumption required for data transmission. They conducted experiments using the iFogSim simulator, demonstrating that their method achieved faster response times and reduced energy consumption compared to substitute methods. In contrast, another paper [18] introduces a novel algorithm named “Fair and Energy Minimized Task Offloading” (FEMTO) specifically designed for Fog computing-enabled IoT networks. This algorithm considers the offloading energy usage, historical average energy of Fog Nodes (FNs), and FN priority, while ensuring fairness in scheduling. FEMTO concludes the optimal transmission power, target FN, and subtask size in a fair and energy-efficient manner. Based on extensive simulations, the proposed algorithm demonstrates greater FN feasibility and minimized energy consumption for work offloading.

Khosroabadi et al. [19] proposed the SCATTER algorithm, which is based on an integrated Fog–Cloud environment, to address the Service Placement Problem (SPP). They introduced a hierarchical Fog–Cloud architecture that clusters Fog nodes into multiple groups, emphasizing the horizontal scalability of the Fog layer. This architecture aims to optimize the utilization of computing resources in the Fog layer. The authors conducted simulations using the iFogSim toolkit and performed experimental evaluations using real hardware, focusing on a smart home application. They compared the SCATTER algorithm with two existing approaches, namely edge-ward and Cloud-only, using Quality of Service (QoS) metrics. The experimental results demonstrated that the SCATTER approach outperformed the edge-ward and Cloud-only approaches in terms of various performance measures. Specifically, the SCATTER approach achieved 42.1% and 60.2% lower application response times, 22% and 27.8% less network usage, 45% and 65.7% less average application loop delays, and 2.33% and 3.2% less energy consumption compared to the edge-ward and Cloud-only approaches, respectively.

In [20], the authors suggested a method for placing incoming modules onto Fog devices that takes into account Quality of Experience (QoE) and energy efficiency. This involves using Fuzzy logic-based approaches and a multi-constraint single objective optimization technique for QoE-aware application mapping. The results indicate that applications with the QoE-aware policy had shorter execution times compared to those without it. Additionally, the simulation results demonstrated that the proposed method helped reduce energy consumption.

Sriraghavendra et al. [21] introduced the DoSP algorithm, which considers the response time of service placement in various layers of a Fog–Cloud architecture. This algorithm makes decisions regarding the placement of modules/services for workflow-based IoT applications. The DoSP algorithm was evaluated using the iFogSim simulator, and the results showed that it outperformed other approaches, such as EdgeWard and Cloud Only, in terms of performance.

Rahbari and Nickray [22] proposed a method for task offloading in mobile Fog computing using a regression and classification tree. Their algorithm, MPCA (Module Placement method by Classification and regression tree Algorithm), selects the optimal Fog modules by checking the power consumption of mobile devices and offloading if it exceeds the usage of Wi-Fi. The authors applied seven parameters to choose the best Fog device, including authentication, confidentiality, integrity, availability, capacity, speed, and cost. They also optimized MPCA using the probability of network resource utilization, called MPMCP. Comparing MPCA and MPMCP with First Fit and local processing methods, the authors claim that the MPMCP approach outperforms them. The advantages and limitations of the techniques used in the existing literature survey are presented in Table 1.

## 3. System Architecture and Computing Models

**Figure 1:**
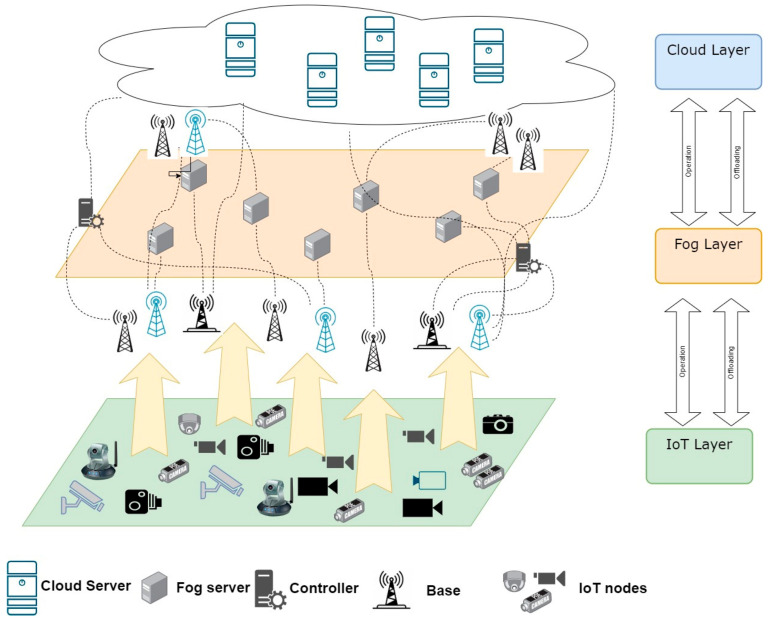
The system architecture.

This section presents the IoT–Fog–Cloud framework, communication model, and computation models across three layers. Figure 1 depicts a general outline of the IoT–Fog–Cloud architecture, which contains three layers. The first layer, referred to as the infrastructure layer, consists of IoT devices responsible for processing tasks either independently or forwarding them to higher levels. The second layer, known as the Fog layer, encompasses multiple Fog devices placed at various geographical locations, offering diverse capabilities. These devices can be underutilized servers, gateway servers, routers, switches, etc. [23]. Additionally, within the second layer, a controller gathers information from the Fog devices, such as available MIPS, host utilization, and network bandwidth. Using this data, the controller determines whether to offload requests to the most suitable Fog nodes or to the Cloud in the third layer. The Fog layer efficiently handles numerous tasks to minimize service delay and energy consumption of application requests [24]. Particularly, the model does not consider offloading between Fog nodes. 

Fog devices possess limited resources in terms of processor, memory, and bandwidth capacity. When developing Fog computing applications, the distributed data stream model is employed. In order to deploy IoT applications in a distributed environment, it is essential to create models for the different application modules that make up the data processing components. This paper adopts the Distributed Data Flow Model (DDF) to accomplish this task [25].

In Fog computing, modules require specific resources for program execution. When these modules are installed in a Fog environment, it is important to ensure that the requested resources do not go beyond the available resources of the respective machine. Hence, within Fog computing, there exist N nodes that serve as Fog sources, with each node possessing a selected capacity. If we denote node *i* of the network as fi, we can represent the following:

(1) $$N=\sum f_{i}$$

(2) $$R_{fi}=\left(CPU_{i};\;RAM_{i\;};Bandwith_{i}\right)\;$$

$R_{fi}$ represents the capacity of node i and also, IoT applications include the M modules, so that:

(3) $$M=\sum m_{i}$$

If we consider m as a module of the application:

(4) $$Re_{mi}=\;\left(CPU_{i};\;RAM_{i\;};Bandwith_{i}\right)$$

$Re_{mi}$ represents the required resources for the module from the program and shows the module mapping to the device with the following (5):

*P*: *M* → *F* (5)

In this map, some restrictions are considered as follows in Equation (6).

(6) $$\forall \left(m_{i};f_{i}\right)\;|\;Re_{mi}<R_{fi};\;\forall f_{i}\in N;\;\forall m_{i}\in M$$

## 4. Proposed Algorithm

This section presents a detailed explanation of our proposed algorithms. The recommended approach, outlined in Algorithm 1 and Algorithm 2, focuses on energy productivity by effectively assigning incoming application modules (tasks) to the most suitable Fog devices. If an IoT device is incapable of executing a task, an agent controller selects the optimal Fog node for task execution based on the result of the multi-classifier machine learning model (MCEETO). We evaluated various individual and ensemble classification techniques to select the highest accuracy to be used in MCEETO. In contrast to individual techniques, ensembles have the capability to amalgamate multiple weak learners into robust learners, resulting in improved accuracy, stability, and robustness [26]. Bagging, boosting, and stacking are three widely recognized ensemble techniques, although there are also other alternatives and ensemble algorithms utilized in practical applications. As stated in [27], here is a brief description of the different bagging, boosting, and stacking ensemble techniques:

- Bagging is an ensemble technique that involves training multiple learners on subsamples of the original data. The predictions from these learners are then combined to generate a representative value, which can be the mean, median, or majority vote for classification tasks, or averaging for regression tasks. The specific choice of the combination depends on the nature of the problem being addressed;
- Boosting algorithms employ a forward stage-wise technique to enhance the performance of weak learners, by iteratively adjusting the weights of training samples that were inaccurately classified or miscalculated. The ultimate outcome of boosting is derived by aggregating the outputs from all iterations, utilizing either a weighted voting scheme for classification or a weighted sum for regression;
- In 1992, Wolpert introduced the concept of stacked generalization, also referred to as stacking. Stacking is a heterogeneous learning approach that trains a model by combining multiple diverse base learners. In contrast to the homogeneous bagging and boosting methods that aggregate learner outputs for final predictions, stacking leverages the unique strengths of various base learners. By utilizing either majority voting or weighted averaging, stacking is expected to outperform individual base learners.

**Figure 2:**
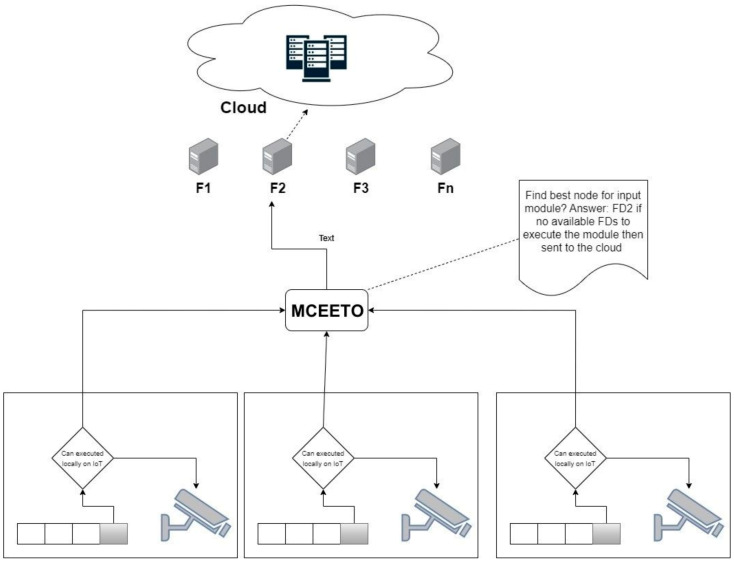
Scheme of the module offloading from IoTs to FDs or Cloud.

The classification model takes into account the characteristics of the task, as well as different information about the Fog nodes and network topology, including node utilization, latency, bandwidth, and more. This process is proved in Figure 2. If there are no available Fog nodes capable of handling the task, it will be offloaded to the Cloud through the Internet. Furthermore, the approach aims to optimize the utilization of Fog devices, ensuring a well-balanced distribution of workloads that prevents both underutilization and overloading of individual Fog devices. The MCEETO pseudo-code can be found in Algorithms 1 and 2.
**Algorithm 1** Intelligent placement strategy (MCEETO)Input: camDeviceList, FogDeviceList, CloudDevice, modulesList Output: module allocation on the most suitable Fog device in energy efficient mannerfor each module in modulesToPlaceList doif camDevice can execute module thenallocatedDevice = camDeviceelse if FogDevice can execute module thenallocatedDevice = findBestFogNodeforProcessingModule (FogDeviceList, module)elseallocatedDevice = CloudDeviceendplace module on allocatedDeviceend

**Algorithm 2** Find best fog node for processing moduleInput: FogDeviceList, module Output: bestFogDeviceIdwhile (True) doseletedFogNode = findbestFognode (trainedclassifier, FogDeviceList, module)if (seletedFogNode can execute module)return selectedFogNodeIdelsego step 2end whileend

The time complexity of our proposed approach is analyzed under two phases: the time complexity of placement request processing, which is handled by Algorithm 1, and the time complexity of selecting a Fog node for the placement of the module covered by Algorithm 2. The time complexity of Algorithm 1 is approximated as O(m) multiplied by the time complexity of the classification (prediction) step that is used in Algorithm 2, considering a machine learning model like the decision tree. Thus, the time complexity of our proposed approach is O(m × d), where ‘m’ is the number of modules and ‘d’ is tree depth.

## 5. Performance Evaluation

### 5.1. Simulation Tool

Simulation tools, such as DEVS (discrete event system specification) [28], SimPy [29], and iFogSim [12], can be applied for simulating scenarios, including Fog-enabled CoT (Cloud of Things). As stated in [12,30], iFogSim is considered the most applicable tool for simulating application environments incorporating IoT, Fog, and Cloud. iFogSim offers several structures that make it well-suited for this purpose:

- It expands upon CloudSim, a widely-utilized tool for simulating Cloud environments, by enhancing its core elements, including the data center and Cloudlets;
- It is the first simulator that integrates IoT objects, such as sensors, by connecting them to Fog nodes and the Cloud using a hierarchical architecture;
- It is highly suitable for studying and assessing different facets of Fog-enabled CoT applications, including latency, mobility, and energy efficiency.

### 5.2. Case Study

In the case study development, the effectiveness of the proposed placement strategy is evaluated through simulation experiments using a modified application based on intelligent surveillance through distributed camera networks [12]. The intelligent surveillance application comprises five main modules: Motion Detector, Object Detector, Object Tracker, PTZ Control, and User Interface. This model is influenced by the research conducted by Hong et al. [31], where they introduced an API for Fog applications and utilized it to design a vehicle tracking system based on CCTV technology. In this surveillance application, real-time video streams from multiple CCTV cameras are received, and the PTZ control in each camera constantly adjusts the PTZ parameters.

### 5.3. Experimental Setup

In order to conduct experiments using the iFogSim toolkit, it is essential to identify the attributes of the physical infrastructure, application modules, application edges, and their corresponding data flow tuples. The characteristics of the hardware nodes included in the physical infrastructure are outlined in Table 2. Each physical node is defined by its MIPS, RAM (in KB), uplink bandwidth (upBW in KB/s), downlink bandwidth (downBW in KB/s), position in the tree-like topology, rate per MIPS ($), busy power, and idle power (in W). Furthermore, each physical node is equipped with an internal physical host, 1M of storage, x86 system architecture, and a Linux operating system. The latency between the Cloud and proxy server is set to 100 ms, the proxy server to a router is 10 ms, and the router to a camera is 5 ms. The study presented in this paper was conducted on a computer running Windows 10 64-bit, with an Intel Core i7 CPU and 8 GB of memory. These used configuration values in Table 2 are commonly used in the related literature.

### 5.4. Performance Metrics

To relate the proposed method with other placement strategies, we used performance metrics, including energy consumption, which refers to the overall energy consumed by various components of the network, such as Fog devices, sensors, gateways, and others. Equation (7) can be used to calculate this energy consumption.

energy = CEC + (CT − LUUT) × HLU (7)

CEC stands for current energy consumption, whereas CT represents the current time. Furthermore, LUUT provides the value of the most recent utilization update time, and HLU signifies the previous operation of the host. At the beginning of the simulation, the energy consumed is set to zero. After running the simulation and obtaining the energy consumed, the simulation time is determined by subtracting the last utilization update time from the current system time, and then multiplying it by the previous host utilization. This result is then added to the current energy consumed. The energy consumption is measured in megajoules.

Network usage = MST/(TL × TS) (8)

In Equation (8), the values of TL and TS represent the total latency and total size of the tuple, respectively. Maximum Simulation Time Shown with MST.

Cost = CC + (CT − LUUT) × RPM × LU × TM(9)

CC stands for the present cost, CT refers to the current time, LUUT indicates the time of the most recent utilization update, RPM represents the MIPS rate per unit, LU symbolizes the previous utilization, and TM represents the total MIPS capacity of the host. The cost of resource allocation contains various costs, including memory, bandwidth, storage space, and processor allocation. At the onset of the simulation, all costs are initialized to zero. Once the simulation runs and the updated values are obtained, the total simulation cost is calculated using Equation (9). The rate of obtaining per million instructions per second, the last system utilization, and the total million instructions per second per host is multiplied by the difference between the current system time and the utilization update time. The obtained value is then added to the current cost of the simulator. It is important to note that the cost value is always a non-negative number.

Execution Time = CT − SST(10)

Equation (10) employs CT as a representation of the current time and SST as an indicator of the simulation’s starting time. After the simulation is completed, the difference between the system’s current time and the simulation’s start time is determined, signifying the elapsed simulation time. This measurement is stated in milliseconds and is computed using the executive clock of the simulator.

### 5.5. Comparison with Other Placement Strategies

To calculate the effectiveness of the proposed placement algorithm, we conducted a comparative analysis between our MECCTO approach and two existing policies, edge-ward, and Cloud-only approaches, implemented within the iFogSim framework. In the edge-ward approach, services are primarily placed closer to the network edge. If edge devices lack sufficient computational resources to handle the services, a hierarchical search is performed to identify appropriate devices at higher levels with the required computational capabilities. Particularly, the edge-ward approach does not reflect factors such as application and task priorities, horizontal connections between devices at the same level, or clustering of Fog nodes. In contrast, the Cloud-only approach places all modules of an application to be run in data centers.

### 5.6. Result and Analysis

In this paper, ensemble machine learning classifier methods such as Bagging, Stacking, and Voting were used to achieve better performance than single-classier models and reduce the spread or dispersion of the predictions. To perform classifier training, a dataset was utilized through Weka Java API. The dataset consists of over 10,000 samples, where each sample represents one simulation cycle and contains runtime attribute information of all nodes, network links, and task attributes. The samples are categorized by the ID of the selected processing node. The dataset was generated based on a similar implementation of Algorithm 1, with the only difference being that in step 9, a deterministic approach was used instead of an intelligent ML classifier. In this approach, the node selection involves iterative loops across all available Fog nodes. For each Fog node, the expected energy consumption, time execution, and network usage are calculated, and the Fog node with the minimum value is selected as the processing node for the task offloading. The prediction accuracy achieved by all the models is shown in Table 3 below:

#### 5.6.1. Energy Consumption

**Figure 3:**
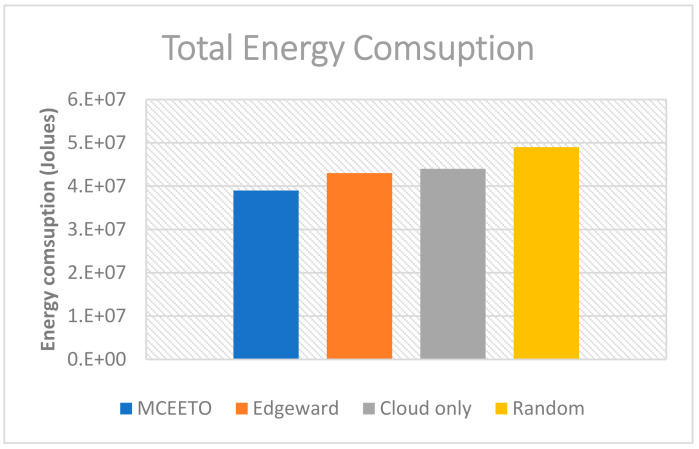
The total energy consumed in joules under various allocation strategies.

The energy consumption of IoT devices, Fog devices, and the Cloud datacenter is calculated using three different strategies: Cloud only, edge-ward, and the proposed MCEETO placement strategy. The total energy consumed is shown in Figure 3. The results demonstrate that the proposed strategy is more energy-efficient compared to the other three strategies, with energy savings of nearly 11.36% compared to Cloud-only and 9.30% compared to the edge-ward strategy.

#### 5.6.2. Network Usage

**Figure 4:**
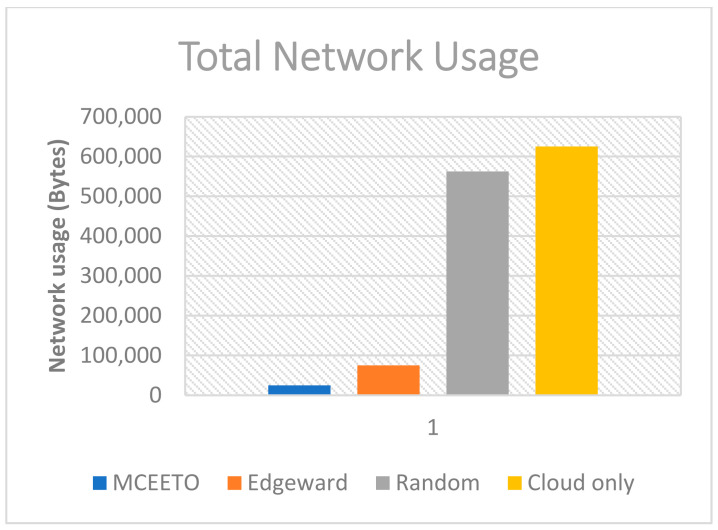
The total network usage under various placement strategies.

Every Internet of Things (IoT) application starts by generating a request from the end-user device. This request is then sent to higher layers for task execution. Each request has a defined size in bytes and is transmitted to a specific target device. The network usage of each request depends on its size and the latency between the starting and target devices. The overall network utilization is calculated by summing up the network usage of each individual request over the entire simulation period. Figure 4 clarifies that the proposed MCEETO algorithm outperformed both Edgeware and Cloud-only strategies, respectively.

#### 5.6.3. Simulation Time

**Figure 5:**
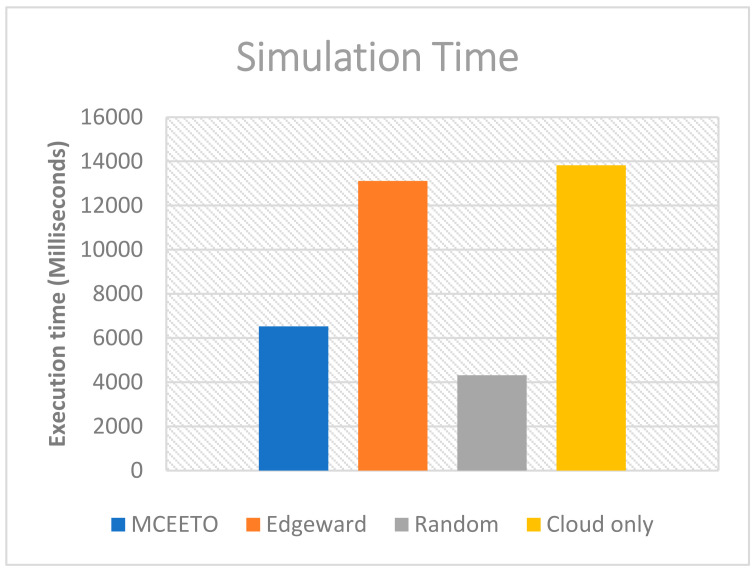
Execution time under various allocation strategies.

The simulation execution time for the three strategies being compared is calculated using Equation (4). Figure 5 illustrates that the MCEETO strategy exhibits the shortest execution time compared to both the edge-ward and Cloud-only strategies. This is attributed to the developments made in the processing node section of our projected strategy, which facilitates a greater portion of task execution in the Fog layer compared to the other strategies.

## 6. Conclusions and Future Works

This paper introduces a groundbreaking Fog computing model and offloading policy to efficiently bring computing power closer to mobile users, addressing challenges in ensuring satisfactory computation performance within the Fog computing environment.

Our study proposes an efficient heuristic algorithm for the service placement problem in Fog–Cloud computing. It prioritizes placing delay-sensitive application services near IoT devices. We compare it with edge-ward, random, and Cloud-only strategies. Unlike Cloud-only and edge-ward approaches causing significant task delay and energy consumption, our algorithm ensures no violation of application deadlines. It optimally utilizes resources in the Fog landscape.

Our approach demonstrates significant energy consumption reduction compared to edge-ward and Cloud-only policies, achieving approximately 11.36% and 9.30% reductions, respectively. Additionally, MCEETO exhibits superior network usage compared to both strategies, with reductions of 67% and 96%, respectively. Future research aims to implement the proposed approach in realistic environments, testing it under various user preferences and application criticality levels within the Fog–Cloud computing paradigm.

## Figures and Tables

**Table 1:** Existing literature surveys with advantages and limitations of used techniques.

Author	Proposed Technique	Advantages/Achievement	Limitations of the Techniques
Kim et al. [13]	Probabilistic method	A considerable amount of energy was saved across various Cloud task requirements.	The stability condition of each network component was not deemed satisfactory.
Shahryari et al. [14]	Genetic algorithm (GA) and particle swarm optimization (PSO)	The proposed algorithm demonstrated superior offloading efficiency in comparison to other algorithms.	Restricted to comparable functionalities of IoT devices.
Wang et al.’s [15]	Gini coefficient	Maximized the aggregate revenue generated by user equipment (UEs).	FCN mobility results in incomplete task migration, causing higher energy consumption and delays.
Cai et al.’s [16]	Lyapunov optimization	The task execution delay and energy consumption at the task node were reduced.	Task queues at the node level were not taken into consideration.
Keshavarznejad et al. [17]	Bees search algorithm	Achieved a more optimal balance between offloading probability and power consumption simultaneously.	The investigation did not encompass the potential for node failure or the deadline for task execution.
Zhang et al. [18]	Fairness metric	Successfully struck a commendable equilibrium between minimal energy consumption and equitable task offloading across Fog Nodes (FNs).	Time-sensitive applications are not supported.
Rahbari and Nickray [22]	Classification and regression tree algorithm	Improved energy efficiency and task latency in comparison to the first-fit (FF) and local mobile strategies.	Prone to failure due to reliance on a centralized decision controller.

**Table 2:** Physical nodes configuration.

	Cloud	Fog Node	IoT Camera
CPU (MIPS)	89,600	6400–9600	500–3200
RAM (KB)	40,000	2000–4000	1000–2000
UpBW (Kb/s)	100	10,000	10,000
DownBW (KB/s)	10,000	10,000	270
BusyPower (W)	103 × 16	107.339	87.53

**Table 3:** Prediction accuracy of different ML methods with latency and energy values.

Accuracy List					
Classifier					Accuracy
Bagging					91.19%
Decision tree					92.93%
Vote					93.55%
RandomForest					94.16%
Stacking					94.54%
	Total Energy (MJ)	Execution Time (MS)	Total Network Usage (MB)	Cost of execution in Cloud ($Million)	
MCEETO	39	6531	0.2	0.7	
Edge-ward	43	13,111	0.6	2	
Random	49	4314	4.5	28	
Cloud only	44	13,819	5	28	

## Data Availability

Not applicable.
